# Expression Levels of miR-99a and miR-143 in Women Diagnosed with Recurrent Pregnancy Loss

**DOI:** 10.3390/jcm15155955

**Published:** 2026-07-30

**Authors:** Konstantina Kouvoutsaki, Eleni Nazou, Despoina Mavrogianni, Emmanouela Liokari, Ismini Anagnostaki, Maria Tzeli, Ioannis Arkoulis, Sofoklis Stavros, Alexandros Rodolakis, Peter Drakakis, Ekaterini Domali

**Affiliations:** 1 Reproductive Biology Laboratory, First Department of Obstetrics and Gynecology, Alexandra Hospital, Medical School, National and Kapodistrian University of Athens, 10559 Athens, Greece; 2 Third Department of Obstetrics and Gynecology, Attikon Hospital, Medical School, National and Kapodistrian University of Athens, 10559 Athens, Greece; nazoueleni@gmail.com (E.N.); isanagnostaki3@gmail.com (I.A.); garkoylis@hotmail.com (I.A.); sfstavrou@yahoo.com (S.S.); pdrakakis@hotmail.com (P.D.); 3 Fertility Institute Athens, 11528 Athens, Greece; meliokari@hotmail.com; 4 Department of Midwifery, Faculty of Health and Caring Sciences, University of West Attica, 12243 Egaleo, Greece; mtzeli@uniwa.gr; 5 First Department of Obstetrics and Gynecology, Alexandra Hospital, Medical School, National and Kapodistrian University of Athens, 10559 Athens, Greece; a.rodolaki@gmail.com (A.R.); k.domali@yahoo.fr (E.D.)

**Keywords:** miRNAs, recurrent pregnancy loss, spontaneous miscarriages, repeated implantation failure

## Abstract

**Background**: Miscarriage is defined as the spontaneous loss of a pregnancy before fetal viability and includes all pregnancy losses occurring from conception until 24 weeks of gestation. Recurrent pregnancy loss (RPL) is traditionally defined as three or more consecutive miscarriages. Emerging evidence suggests that both miR-99a and miR-143 may serve as promising biomarkers for recurrent pregnancy loss. **Methods**: A total of 42 women of reproductive age (18–45 years) were enrolled in this study. The control group consisted of 13 women with no history of recurrent miscarriage, whereas the study group included 29 women diagnosed with spontaneous miscarriages/recurrent implantation failure. **Results**: Both miR-99a and miR-143 showed significantly lower relative expression in women with recurrent miscarriage/recurrent implantation failure compared with the control group. **Conclusions**: Despite the relatively small sample size, statistically significant differences in the expression of miR-99a and miR-143 were observed between the case and control groups, with lower relative expression of both miRNAs in the case group. These findings suggest that both microRNAs may be associated with molecular pathways involved in reproductive failure. Furthermore, investigating the expression patterns of miR-99a and miR-143 may contribute to a better understanding of the molecular mechanisms underlying recurrent miscarriage/recurrent implantation failure. Such insights may facilitate the development of genetic risk profiles for women with unexplained pregnancy loss and implantation failure, as well as support the identification of potential therapeutic targets. Further studies are required to determine their potential value as molecular markers and to clarify their functional relevance in reproductive failure.

## 1. Introduction

Miscarriage is defined as the spontaneous loss of a pregnancy before fetal viability and represents one of the most common complications of early pregnancy [1,2]. While sporadic miscarriage affects a substantial proportion of couples and is most frequently associated with chromosomal abnormalities, recurrent pregnancy loss (RPL) represents a complex reproductive disorder characterized by repeated pregnancy failure [3]. Recurrent miscarriage and recurrent implantation failure (RIF) are considered related but distinct clinical entities [4]. Recurrent miscarriage refers to repeated spontaneous pregnancy losses, whereas RIF describes the failure to achieve a clinical pregnancy following repeated embryo transfer attempts despite the availability of embryos considered suitable for transfer. Due to the absence of reliable diagnostic or prognostic markers, the molecular mechanisms underlying these conditions remain an area of active investigation.

MicroRNAs (miRNAs) are small, single-stranded, non-coding RNA molecules consisting of approximately 20–23 nucleotides [5]. They regulate gene expression at the post-transcriptional level [6] by inhibiting mRNA translation and play an important role in regulating the properties of human blastocysts and embryonic development. Dysregulation of miRNAs during early fetal development affects cell differentiation and the implantation process [7]. Furthermore, differences in miRNA expression have been observed between first- and third-trimester trophoblasts [8], highlighting their involvement in placental development.

Among the numerous miRNAs that have been investigated, miR-99a and miR-143 have attracted particular interest because of their potential association with recurrent pregnancy loss. miR-99a, together with miR-99b and miR-100, belongs to the miR-99 family. It has been shown to be downregulated in several malignancies, including ovarian [9], lung [10], and bladder cancer [11]. Moreover, miR-99a induces apoptosis by targeting ESR1 (Estrogen Receptor 1) in endometrial cancer [12] and has also been proposed as a promising biomarker for breast cancer diagnosis [13].

miR-143 is located on chromosome 5q33 in the human genome [14]. Due to its close genomic proximity to miR-145, it has been suggested that both miRNAs are co-transcribed as a bicistronic primary transcript [15]; consequently, they are frequently investigated together. miR-143 plays an important role in cardiac morphogenesis and has been identified as one of the major miRNAs involved in the differentiation of mouse embryonic stem cells into cardiac progenitor cells [15]. In addition, miR-143 has been implicated in carcinogenesis and tumor progression [16]. Zhang et al. reported the upregulation of miR-143 in a hepatocellular carcinoma model [17]. Furthermore, altered expression of miR-143 has been reported in different stages of cancer [16], while Hossian et al. demonstrated that its combined expression with miR-506 reduces angiogenesis in lung cancer cell lines [18].

## 2. Materials and Methods

### 2.1. Patient Inclusion and Sample Size

The present case–control study was conducted between January 2023 and July 2024 at the First Department of Obstetrics and Gynecology, “ALEXANDRA” General Hospital, Medical School, National and Kapodistrian University of Athens, Greece.

A total of 42 women of reproductive age (18–45 years) were enrolled in the study. The control group consisted of women without a history of recurrent reproductive failure, including participants with no previous miscarriage or with a single sporadic miscarriage, who had subsequently achieved at least one successful pregnancy either naturally or through assisted reproductive technology (ART). These criteria were selected to exclude women with recurrent reproductive failure while allowing inclusion of women with a history of isolated pregnancy loss, which is relatively common in the general population. The study group consisted of 29 women with recurrent reproductive failure, including women with spontaneous miscarriages/recurrent implantation failure. Among the participants with recurrent miscarriage, the number of pregnancy losses varied, with some women having experienced more than three spontaneous miscarriages. Given the overlap in the proposed molecular mechanisms underlying recurrent miscarriage and recurrent implantation failure, particularly regarding impaired implantation, altered endometrial receptivity, and dysregulated inflammatory pathways, these conditions were analyzed collectively as reproductive failure phenotypes. Nevertheless, the potential clinical heterogeneity between these reproductive failure phenotypes is acknowledged as a limitation of the present study.

The inclusion criteria were as follows: women aged 25–45 years, a normal hormonal profile according to the World Health Organization (WHO) guidelines, and a regular menstrual cycle of 21–35 days. All participants were healthy, with no history of cancer, eclampsia, or primary ovarian insufficiency, and represented different ethnic backgrounds. Furthermore, none of the participants was pregnant at the time of sample collection or receiving hormonal therapy. Women with recurrent pregnancy loss who had achieved at least one successful full-term pregnancy were excluded from the study.

The study was conducted in accordance with the Declaration of Helsinki and was approved by the Institutional Ethics Committee of “ALEXANDRA” General Hospital, Medical School, National and Kapodistrian University of Athens (Protocol No. 4262/19.01.2022). Written informed consent was obtained from all participants prior to their inclusion in the study and for the publication of the study findings.

### 2.2. RNA Isolation, Reverse Transcription, and Quantitative Real-Time PCR

Peripheral whole blood samples were collected from all participants, and total RNA was isolated from whole blood samples using the Monarch Total RNA Miniprep Kit (New England Biolabs, Hitchin, UK), according to the manufacturer’s instructions. The extracted RNA samples were stored at −80 °C until further analysis.

RNA was reverse-transcribed into complementary DNA (cDNA) using 1× LunaScript RT SuperMix (New England BioLabs) in 20 μL reaction volumes under the following cycling conditions: 25 °C for 2 min, 55 °C for 10 min, and 95 °C for 1 min. The assay was designed to quantify mature miR-99a and miR-143. The synthesized cDNA samples were subsequently stored at −20 °C.

Quantitative real-time PCR (qPCR) was performed using the Luna Universal qPCR Master Mix (NEB #M3003) (New England Biolabs, Hitchin, UK). The G6PD gene was used as the endogenous reference gene for normalization of miR-99a and miR-143 expression levels according to the experimental protocol applied in the present study. ΔCt values were calculated as Ct_target − Ct_reference, where Ct_target represents the threshold cycle of the target miRNA and Ct_reference that of the endogenous reference gene (G6PD). Relative expression levels of miR-99a and miR-143 were subsequently determined using the 2^−ΔΔCt^ method.

Real-time PCR amplification was carried out using the LightCycler^®^ 480 Real-Time PCR System (Roche, Basel, Switzerland) (Table 1). The reported amplicon sizes correspond to the cDNA-derived qPCR products generated during reverse transcription and do not represent the length of the native mature miRNAs.

### 2.3. Statistical Analysis

Differences in gene expression between the control group and the recurrent pregnancy loss group (cases) were evaluated using an independent-samples *t*-test. The Kolmogorov–Smirnov test was used to assess data normality, while Levene’s test was applied to evaluate the homogeneity of variances.

To investigate the main effects and potential interactions of group, age, body mass index (BMI), and smoking status on miR-99a and miR-143 expression levels, a two-way analysis of variance (Two-Way ANOVA) with interaction terms was performed.

All statistical analyses were conducted using SPSS (Statistical Package for the Social Sciences) Version 31.0.0, and statistical significance was defined as *p* < 0.05.

## 3. Results

**Figure 1:**
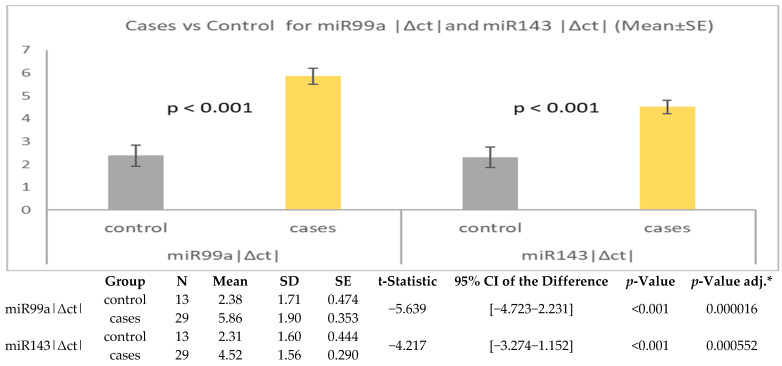
Comparison of miR-99a and miR-143 expression between control and case group. The assumptions for the application of the parametric test were assessed and confirmed, as the data demonstrated both normal distribution and homogeneity of variance between the groups. Therefore, an independent samples t-test was applied, revealing statistically significant differences between the two groups for the variables miR-99a (*p* = 0.038), miR-99a (*p* < 0.001), and miR-143 (*p* < 0.001). * Adjustment for multiple comparisons: Bonferroni.

**Figure 2:**
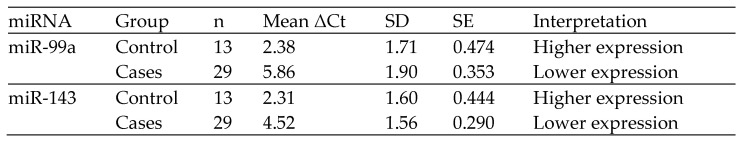
Descriptive statistics of ΔCt values for miR-99a and miR-143 in the control and case groups. Mean ΔCt values, standard deviation (SD), and standard error (SE) are presented. Since ΔCt was calculated as Ct_target−Ct_reference, higher ΔCt values indicate lower relative expression. Accordingly, the case group exhibited higher mean ΔCt values for both miRNAs than the control group.

Forty-two samples were obtained from women of reproductive age (n = 42) and analyzed with respect to age, body mass index (BMI), and smoking status (Table 2). Mean ΔCt values for miR-99a and miR-143 are presented in Table 2. Since ΔCt values were calculated as Ct_target − Ct_reference, higher ΔCt values correspond to lower relative miRNA expression. The case group exhibited higher mean ΔCt values for both miR-99a (5.86 vs. 2.38) and miR-143 (4.52 vs. 2.31) compared with the control group, indicating lower relative expression of both miRNAs in women with recurrent pregnancy loss/recurrent implantation failure (Figure 1 and Figure 2).

**Figure 3:**
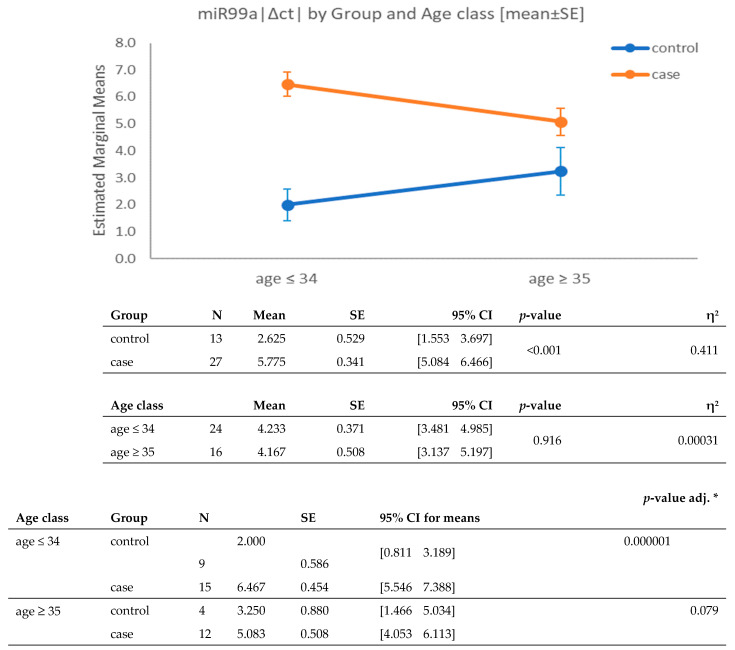
miR-99a expression in both groups divided into two subgroups under and over 35 years old. A statistically significant difference was observed between the two groups (*p* < 0.001), with the miscarriage group (cases) exhibiting higher miR-99a ΔCt values, corresponding to lower relative miR-99a expression. The effect size (η^2^ = 0.411) indicates a strong effect of group on miR-99a expression. miR-99a expression did not differ significantly according to age group (*p* = 0.916). The interaction effect (Group × Age class) was statistically significant (*p* = 0.043). Confidence Intervals of 95% represent the intervals for the subgroup means. *p*-values represent the pairwise comparisons between control and case groups within each age class, adjusted using the Bonferroni correction. The two groups differed significantly only within the age group <34 years (adjusted *p*-value < 0.001). As illustrated in the figure, the difference between the groups was more pronounced at younger ages, whereas it became less evident with increasing age. * Adjustment for multiple comparisons: Bonferroni.

A statistically significant interaction between group and age was observed for miR-99a expression (*p* = 0.043), indicating that the association between age and miR-99a levels differed between the two groups. Specifically, in the recurrent pregnancy loss/recurrent implantation failure group, miR-99a ΔCt values showed a decreasing trend with increasing age, whereas in the control group, ΔCt values tended to increase with age. As illustrated in Figure 2, the difference in miR-99a expression between groups appeared more pronounced at younger ages, while this difference was attenuated at older ages. Post hoc analysis (Figure 3) demonstrated that a statistically significant difference between the two groups was observed only among participants younger than 34 years (adjusted *p*-value < 0.001). At older ages, no statistically significant difference between the groups was detected.

**Figure 4:**
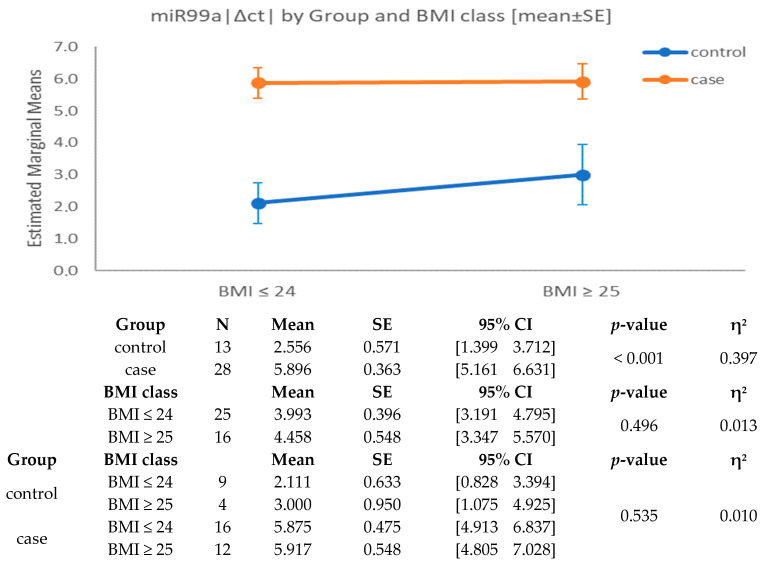
miR-99a expression in both groups divided into two subgroups according to their BMI. The main effect of group was statistically significant (*p* < 0.001), miR-99a ΔCt value was significantly higher in the miscarriage group (cases), corresponding to lower relative miR-99a expression, independently of BMI. In contrast, miR-99a expression did not appear to be associated with BMI (*p* = 0.496), regardless of group assignment. Finally, no statistically significant interaction between group and BMI was observed (*p* = 0.535), which is also reflected graphically by the parallel trends of the two lines (blue and orange). This indicates that the lack of association between BMI and miR-99a expression is consistent in both the control and miscarriage groups. Alternatively, the difference in miR-99a expression between controls and cases remains comparable across BMI categories.

**Figure 5:**
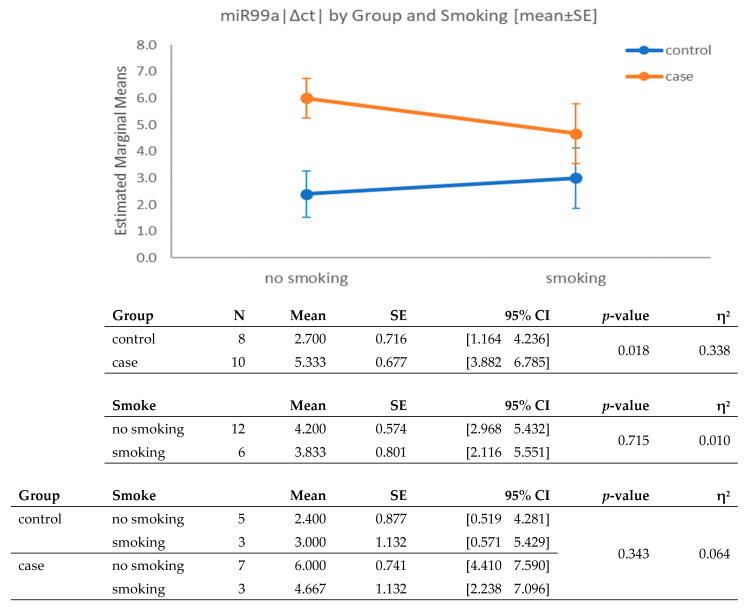
miR-99a expression in both groups divided into two subgroups according their smoking habits. The main effect of group was statistically significant (*p* = 0.018), indicating that the mean miR-99a ΔCt value was significantly higher in the miscarriage group (cases), corresponding to lower relative miR-99a expression, independently of smoking status. In contrast, miR-99a expression did not appear to be associated with smoking status (*p* = 0.715), regardless of group assignment. Finally, no statistically significant interaction between group and smoking status was observed (*p* = 0.343), suggesting that smoking status does not modify the difference in miR-99a expression between the control and miscarriage groups.

As shown in Figure 4, the main effect of group on miR-99a ΔCt values was statistically significant (*p* < 0.001), indicating significantly higher ΔCt values in the case group than in the control group, independently of BMI. Since higher ΔCt values correspond to lower relative expression, these findings indicate lower miR-99a expression in the case group. In contrast, miR-99a ΔCt values did not show evidence of association with BMI (*p* = 0.496), and no significant interaction between either group and BMI was observed. Similarly, no significant association between miR-99a expression and smoking status was identified (*p* = 0.715), and no statistically significant interaction between both groups and smoking status was detected (*p* = 0.343) (Figure 5).

**Figure 6:**
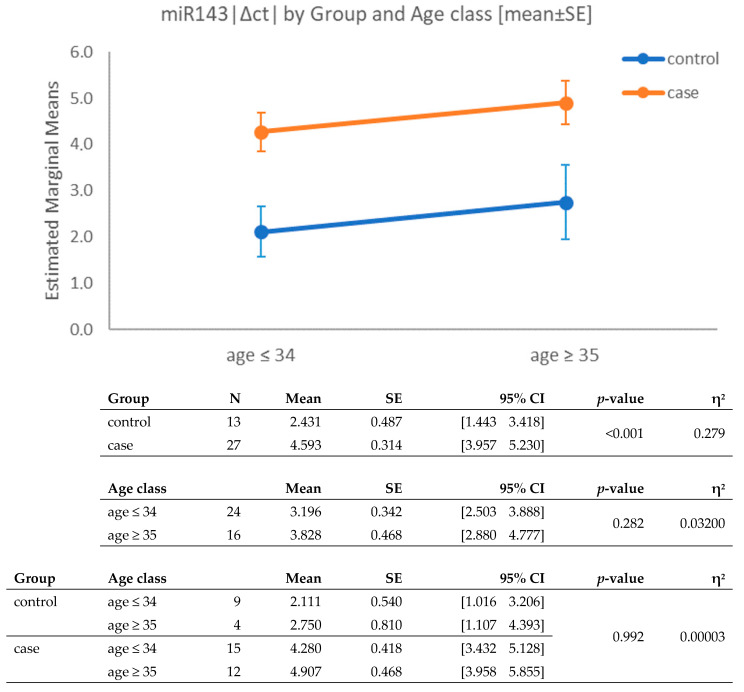
miR-143 expression in both groups divided into two subgroups under and over 35 years old. The main effect of group was statistically significant (*p* < 0.001), indicating that the mean miR-143 ΔCt value was significantly higher in the miscarriage group (cases), corresponding to lower relative miR-143 expression, independently of age group. In contrast, miR-143 expression did not appear to be associated with age (*p* = 0.282), regardless of group assignment. Finally, no statistically significant interaction between group and age was observed (*p* = 0.992), which is also reflected graphically by the parallel trends of the two lines (blue and orange). This indicates that the lack of association between age and miR-143 expression is consistent in both the control and miscarriage groups. Alternatively, the difference in miR-143 expression between controls and cases remains comparable across age groups.

**Figure 7:**
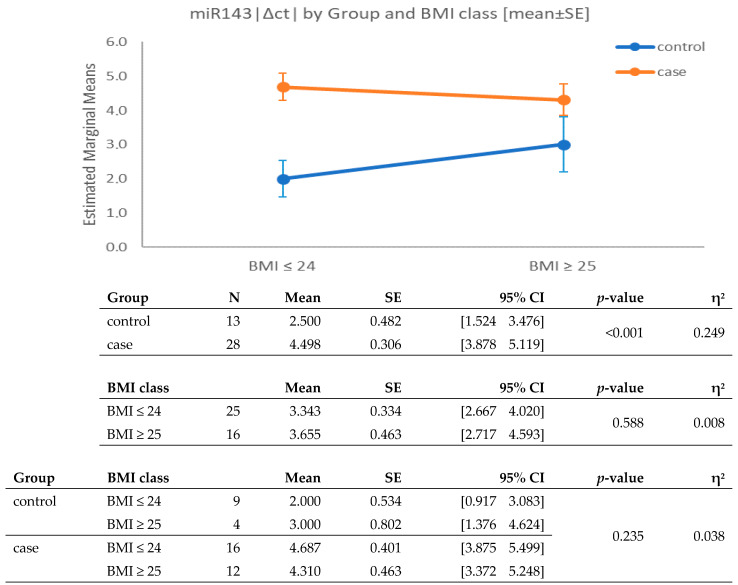
The main effect of group was statistically significant (*p* < 0.001), miR-143 ΔCt value was significantly higher in the miscarriage group (cases), corresponding to lower relative miR-143 expression, independently of BMI category. In contrast, miR-143 expression did not appear to be associated with BMI (*p* = 0.588), regardless of group assignment. Finally, no statistically significant interaction between group and BMI was observed (*p* = 0.235). This indicates that the lack of association between BMI and miR-143 expression is consistent in both the control and miscarriage groups.

**Figure 8:**
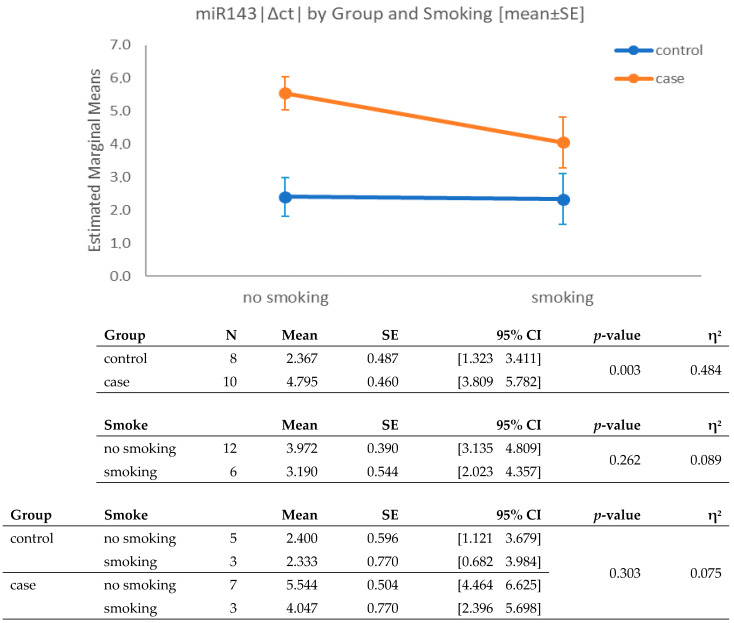
miR-143 expression in both groups divided into two subgroups according their smoking habits. The main effect of group was statistically significant (*p* = 0.003), indicating that the mean miR-143 ΔCt value was significantly higher in the miscarriage group (cases), corresponding to lower relative miR-143 expression, independently of smoking status. In contrast, miR-143 expression did not appear to be associated with smoking status (*p* = 0.262), regardless of group assignment. Finally, no statistically significant interaction between group and smoking status was observed (*p* = 0.303), suggesting that smoking status does not modify the difference in miR-143 expression between the control and miscarriage groups.

In contrast to miR-99a, miR-143 ΔCt values were not significantly associated with age (*p* = 0.282), and no interaction between group and age was observed (*p* = 0.992) (Figure 6). Likewise, miR-143 expression did not appear to be influenced by BMI (*p* = 0.588), with no significant interaction between BMI and both groups identified (*p* = 0.235), suggesting that the absence of a BMI-related effect was consistent across both groups (Figure 7). Finally, miR-143 ΔCt values were not significantly associated with smoking status (*p* = 0.262), and no significant interaction between the groups and smoking was observed (*p* = 0.303) (Figure 8).

## 4. Discussion

Reproductive failure, including recurrent pregnancy loss (RPL) and recurrent implantation failure (RIF), remains a challenging clinical condition affecting women of reproductive age. Although several contributing factors have been identified, including genetic [19,20], structural [21], endocrine [21,22], immunological, and thrombotic abnormalities [23], a substantial proportion of cases remain unexplained. Increasing evidence suggests that alterations in molecular regulatory mechanisms may contribute to impaired implantation and pregnancy maintenance. It is estimated that the incidence of pregnancy loss following natural conception ranges from 25% to 40% [24]. Among women with recurrent miscarriage, implantation failure is considered to account for approximately 75% of cases [25]. Moreover, nearly half of all embryo implantations fail to result in a successful pregnancy. Previous studies have demonstrated that recurrent miscarriage and recurrent implantation failure share several common underlying mechanisms, primarily involving embryo quality, endometrial receptivity, immune response, and the maternal genetic profile.

Infertility affects approximately 13–15% of couples worldwide [26]. Consequently, considerable research has focused on identifying factors that could improve pregnancy rates and increase the likelihood of a successful outcome. MicroRNAs regulate numerous biological processes, including cell growth, differentiation [27], and placental development [28,29], through their involvement in cell migration, invasion, angiogenesis, and apoptosis. Furthermore, miRNAs have emerged as potential molecular indicators of pregnancy-related complications due to their involvement in placental development and regulation of key cellular processes [30].

According to Geng et al., miR-99a is expressed at lower levels in women with polycystic ovary syndrome (PCOS) than in healthy controls [31], suggesting its potential value as a diagnostic biomarker for PCOS. miR-99a has also been proposed as a promising biomarker for recurrent pregnancy loss. A study investigating the association between miR-99a and transforming growth factor-β1 (TGF-β1) expression and the serum levels of β-human chorionic gonadotropin (β-hCG), progesterone, and estrogen in 70 women diagnosed with early spontaneous abortion demonstrated significantly elevated serum miR-99a expression [32]. Furthermore, miR-99a expression was negatively correlated with β-hCG, progesterone, and estrogen levels in these patients.

In addition, miR-99a has been associated with endometriosis [33] and has been reported to be upregulated in the endometrium of patients with recurrent implantation failure undergoing in vitro fertilization (IVF) [34]. miR-99a has also been shown to interact with members of the interleukin-6 (IL-6) family, particularly leukemia inhibitory factor (LIF) and IL-6. Previous studies have demonstrated that miR-99a suppresses IL-6 expression [35] and regulates endometrial responsiveness to LIF, thereby influencing implantation success [36].

Similarly, increased miR-143 expression has been detected in cervical cells obtained from pregnant women who subsequently experienced preterm birth [37], whereas reduced miR-143 expression has been associated with fetal macrosomia [38,39]. In addition, miR-143 has been implicated in several cellular processes, including cell proliferation, adhesion, invasion, and apoptosis [40]. Elevated serum levels of miR-143 have also been reported in women with endometriosis [41], whereas its expression is reduced in eutopic endometrial tissue compared with ectopic endometrial tissue [42]. Shi Tian et al. concluded that uterine miR-143 contributes to successful blastocyst implantation in rats through the regulation of the leukemia inhibitory factor receptor (LIFR) [43]. In humans, miR-143 has also been shown to suppress IL-6 expression [44]. Although direct evidence supporting the involvement of miR-143 in the implantation process remains limited, accumulating evidence suggests that it contributes to the molecular and cellular environment of the uterus during implantation [43].

Age, modern lifestyle, smoking, and dietary habits have all been associated with reduced fertility in women of reproductive age. Therefore, these parameters were taken into consideration during the design and implementation of the present study.

With regard to age, miR-99a expression did not differ significantly across age groups (*p* = 0.916), whereas miR-143 expression was not influenced by age (*p* = 0.282), irrespective of group. Furthermore, no statistically significant interaction between group and age was observed (*p* = 0.992), indicating that the absence of an age-related effect on miR-143 expression was consistent across both the control and recurrent miscarriage groups.

Similarly, no statistically significant interaction between group and BMI was identified (*p* = 0.535), suggesting that the lack of a BMI effect on miR-99a expression was consistent in both groups. In other words, the differences in miR-99a expression between cases and controls remained similar regardless of BMI category. The main effect of group was statistically significant (*p* = 0.001), miR-143 ΔCt values were significantly higher in the recurrent miscarriage group, corresponding to lower relative miR-143 expression, irrespective of BMI.

Likewise, irrespective of smoking status, the main effect of group remained statistically significant (*p* < 0.018), indicating significantly higher miR-99a ΔCt values, and therefore lower relative miR-99a expression, in the recurrent miscarriage group. Similarly, the main effect of group was statistically significant for miR-143 expression (*p* = 0.003), demonstrating higher miR-143 ΔCt values, corresponding to lower relative expression, in women with recurrent miscarriage, regardless of smoking status. In contrast, miR-143 expression was not associated with smoking status (*p* = 0.262), and no statistically significant interaction between group and smoking was observed (*p* = 0.303).

Although the present study included a relatively small number of participants, differential expression of miR-99a and miR-143 was observed in women with reproductive failure compared with controls, with lower relative expression of both miRNAs in the case group. These findings suggest that altered regulation of these miRNAs may be associated with the molecular processes underlying recurrent pregnancy loss and recurrent implantation failure. Nevertheless, a definitive association between the differential expression of miR-99a and miR-143 and reproductive health risk factors has not yet been fully established.

Interleukin-6 (IL-6) is a key mediator of inflammatory signaling and has been associated with processes relevant to reproductive function, including immune regulation and placental development. Alterations in IL-6-related pathways have also been linked to oxidative stress and impaired tissue homeostasis, mechanisms that may contribute to pregnancy complications [45,46,47,48,49]. Emerging evidence suggests that miRNAs, including miR-99a and miR-143, may participate in the regulation of IL-6-associated signaling pathways. Therefore, the reduced relative expression of these miRNAs observed in the present study may reflect alterations in inflammatory regulatory networks involved in reproductive failure [44,50].

Considering the involvement of IL-6 in inflammation and oxidative stress-related processes, the reduced relative expression of miR-99a and miR-143 observed in the present study may reflect, at least in part, dysregulation of molecular networks associated with these pathways. Although the exact mechanisms remain to be elucidated, these findings support a possible role of miR-99a and miR-143 in the molecular landscape of recurrent pregnancy loss.

Given their regulatory involvement in inflammatory and oxidative stress-related pathways, miR-99a and miR-143 may represent potential candidate biomarkers for recurrent pregnancy loss, although this possibility requires further validation. In the present study, reduced relative expression of these microRNAs was associated with recurrent implantation failure and recurrent pregnancy loss, suggesting their potential contribution to the underlying molecular mechanisms involved in these conditions.

Although recurrent miscarriage and recurrent implantation failure represent distinct clinical conditions, both involve complex interactions between embryo competence, endometrial receptivity, immune regulation, and maternal molecular factors. Therefore, the combined analysis of these phenotypes may provide insights into shared molecular alterations associated with reproductive failure. Nevertheless, future studies including larger cohorts should evaluate these conditions separately to determine whether specific miRNA signatures characterize each phenotype.

The present study has several limitations, including the relatively small sample size, the single-center design, and the clinical heterogeneity of the study population. In the present cohort, women with recurrent miscarriage and recurrent implantation failure were analyzed collectively, as these reproductive failure phenotypes may overlap clinically. Several participants had a history of previous pregnancy losses followed by assisted reproductive procedures and subsequent implantation failure, making a strict classification into mutually exclusive recurrent miscarriage and recurrent implantation failure subgroups challenging. Therefore, separate analyses of these distinct reproductive failure phenotypes could not be reliably performed within the current sample size.

Additionally, although relevant clinical characteristics such as age, body mass index (BMI), and smoking status were considered, other potential factors associated with reproductive failure could not be fully evaluated. These factors may include genetic, endocrine, immunological, thrombotic, and structural abnormalities that may influence reproductive outcomes. Therefore, the contribution of these factors to the observed miRNA expression patterns cannot be completely excluded. Future studies involving larger and more clinically characterized cohorts, including well-defined reproductive failure phenotypes and comprehensive evaluation of potential confounding factors, are required to further investigate the association between miRNA expression patterns and the molecular mechanisms underlying recurrent pregnancy loss and recurrent implantation failure.

## 5. Conclusions

Despite the relatively small sample size, our findings demonstrate differential expression of miR-99a and miR-143 in women with recurrent pregnancy loss and/or recurrent implantation failure compared with controls, with lower relative expression of both miRNAs observed in the case group These results suggest that these microRNAs may be involved in molecular pathways associated with reproductive failure. Although further studies with larger cohorts and functional validation are required, the present findings support the potential involvement of miR-99a and miR-143 as candidate molecular markers and provide a basis for further investigation of their role in recurrent pregnancy loss.

## Figures and Tables

**Table 1:** Primer sequences for RT-PCR and size area.

PRIMER	SEQUENCING	AMPLICON
G6PD	Forward: 5′-GGACCTGACCTACGGCAACAGATA-3′ Reverse: 5′-GCCCTCATACTGGAAACCC-3′	256
miR-99a	Forward 5′-TATTAATAGGGGGCCCATGC-3′ Reverse 5′-ATTGTTGAACGGCACTGTGT-3′	81
miR-143	Forward 5′-CTTTCTTCTGCCACTCCTCCT- 3′ Reverse 5′-GAAGGGCTTCAGAAT TCCG-3′	106

**Table 2:** Demographic characteristics of participants divided in two groups, control group and study group.

	*Control Group*	*Case Group*	*p-Value*
*Age*	31.21 ± 4.54	33.96 ± 5.46	0.084
*BMI*	24.54 ± 4.71	23.96 ± 4.32	0.705

## Data Availability

The original contributions presented in this study are included in the article. Further inquiries can be directed to the corresponding author(s).

## Abbreviations

The following abbreviations are used in this manuscript:

RIF	Repeated implantation failure
RPL	recurrent pregnancy loss
BMI	Body mass index
LIF	Leukemia Inhibitory Factor
